# Extracellular vesicles as key biomarkers in COVID-19: insights into disease severity and mortality through CD86 and other immune markers

**DOI:** 10.3389/fimmu.2026.1703062

**Published:** 2026-03-23

**Authors:** Lou Soret, Coralie L. Guerin, Joseph Roux de Bezieux, Katharine Rus, Léa Guyonnet, Georges Abi Abdallah, Nicolas Gendron, Anne Godier, Diane Zlotnik, Alexandre G. Lellouch, Annick Viguier, Jean-Luc Diehl, Pascale Gaussem, Aurélien Philippe, David M. Smadja

**Affiliations:** 1 University Paris Cité, Inserm Unité mixte de recherche (UMR)-S970, Paris Cardiovascular Research Center, Team Endotheliopathy and Hemostasis Disorders, Paris, France; 2 Hematology Department, Assistance Publique - Hôpitaux de Paris (AP-HP), Centre Université Paris Cité, Georges Pompidou European Hospital, Paris, France; 3 Institut Curie, Cytometry and Extracellular Vesicles Platforms, Paris, France; 4 Department of Anaesthesiology and Critical Care, Assistance Publique - Hôpitaux de Paris (AP-HP), Centre Université Paris Cité, Georges Pompidou European Hospital, Paris, France; 5 Cedars-Sinai Medical Center, Los Angeles, CA, United States; 6 Medical Intensive Care Department, Assistance Publique - Hôpitaux de Paris (AP-HP), Centre Université Paris Cité, Georges Pompidou European Hospital, Paris, France

**Keywords:** CD86, COVID-19, extracellular vesicles, nanoparticle tracking analysis, SARS-CoV-2, videodrop

## Abstract

This study explores the concentration, size, and epitope profiles of extracellular vesicles (EVs) as biomarkers for COVID-19 outcomes. CD86 stood out as a key predictor of mortality, suggesting that EV profiling could help assess disease severity and guide treatment decisions.

**Background:**

SARS-CoV-2 infection triggers a complex array of immune and vascular responses. Extracellular vesicles (EVs) have emerged as critical players in the disease’s progression and potential biomarkers for assessing severity.

**Aim:**

To explore the concentration, size, and epitope profiles of EVs in COVID-19 patients and correlate these findings with clinical outcomes.

**Methods:**

We analyzed EVs from 80 COVID-19 patients (critical or non-critical). EV concentration and size were measured using Nanoparticle Tracking Analysis (NTA) and Videodrop, while antigen expression was assessed via a 37-marker MACSPlex bead assay.

**Results:**

Our findings reveal a significant elevation in circulating EV concentration in critical COVID-19 patients as measured by Videodrop (*p* = 0.007), though not by NTA (*p* = 0.063), highlighting method-specific sensitivities. Key EV surface markers, including CD86, CD8, CD326, CD209, and CD9, were significantly higher in critical patients, while CD19 was reduced. Kaplan-Meier survival analysis showed that higher expression levels of several EV markers, including CD86, were associated with decreased survival. Among these, CD86 emerged as the most potent independent predictor of in-hospital mortality, regardless of inflammatory status.

**Conclusion:**

This study highlights the importance of CD86-expressing EVs as biomarkers for COVID-19 severity and mortality, suggesting that EV profiling could inform personalized therapies for severe cases.

## Introduction

Severe acute respiratory syndrome coronavirus-2 (SARS-CoV-2), the causative agent of COVID-19, induces an infection with complex response patterns that involve coagulopathy and activation of many vascular and immune cell types (1–4). In this context, extracellular vesicles (EVs) have garnered significant attention for their potential role in the disease’s progression and as possible biomarkers or therapeutic targets (5–15). EVs can derive either from the virus-infected cells or from the host immune cells (16). According to different studies, EVs function as a double-edged sword by facilitating viral spreading and immune evasion and tissue damage but also by inducing immune response, regulating viral infection and tissue repair (17, 18). Seiko Carvalho Tahyra et al. showed that EVs contribute notably to heighten hypercytokinemia and interfered in the innate and adaptive immune response to COVID-19 (19). EVs are key players in the mediation and regulation of multiple pathophysiological processes, including innate and adaptive immunity (20). EVs are defined as particles released from cells, delimited by a lipid bilayer, which cannot replicate on their own. The International Society for Extracellular Vesicles (ISEV) separated two groups of EVs based on their diameter: small EVs, smaller than 200 nm, and large EVs, i.e. over 200 nm in size. Noteworthy, the measured diameter is related to the method used (21, 22). The concentration and size of EVs are studied by interferometry methods, such as nanoparticle tracking analysis (NTA), the reference method, or Videodrop, a new approach. The detection of EVs in a sample generally uses the surface expression of tetraspanins by flow cytometry. Indeed, tetraspanins (CD9, CD63, and CD81) are abundantly expressed by many subtypes of EVs, including exosomes, microvesicles and apoptotic bodies (23–25), even if not all EVs express them.

During COVID-19, Barberis et al. identified the presence of SARS-CoV-2 RNA in the exosomal cargo, which suggests that the virus might use the endocytosis route to spread infection (6). Recently, Balbi et al. reported that circulating EVs display enhanced procoagulant activity in COVID-19 and therefore might act as clotting initiation agents to contribute to the disease severity (26). Moreover, large and small extracellular vesicles explored by flow cytometry showed lower levels of CD4+, CD19+ and CD146+ EVs in severe COVID-19 infection, compared to healthy donors (27). Another study highlighted platelet-EVs (CD41+/AnnexinV+ or CD41a+/CD31+) as biomarkers of COVID-19 severity (8, 28). Moreover, Puhm et al. considered platelet-EVs as valuable biomarkers acting as predictors of and contributors to COVID-19 (29). Finally, Mezine et al. highlighted the potential interest of detecting EVs expressing E-selectin (CD62) to discriminate COVID-19 patients at the time of hospital admission and identify individuals with higher risk of fatal outcome (30).

The aim of our study was to investigate how EV concentration and size, measured by two interferometry methods, as well as their epitope profiles, might vary in COVID-19 patients with different levels of disease severity, and how these factors correlate with in-hospital mortality.

## Materials and methods

### Study design and population

The study was monocentric cross-sectional, including adult (≥18 years old) COVID-19 hospitalized patients in the French European Georges Pompidou hospital between March 13 and April 10, 2020. The study was performed in accordance with the Declaration of Helsinki. All patients included, or their trusted relatives, signed a written consent form at the time of enrollment (NCT04624997). All included patients had a diagnosis of SARS-CoV-2 infection confirmed by a RT-PCR test on nasopharyngeal swab samples (Allplex™ 2019-nCoV Assay, Seegene, SK) as previously described (31). Patients were classified according to World Health Organization (WHO) guidance as non-critical (i.e., requiring oxygen supplementation WHO score range 5–6) or critical (i.e., requiring mechanical ventilation; WHO score range 7–9) based on their clinical condition on the day of their hospitalization (32). All non-critical patients (n=40) were initially admitted in medical wards and all critical patients (n=40) were immediately treated in intensive care unit following their admission. Patient characteristics, including age, sex, comorbidities and treatment at admission, were recorded in the REDCAP database (Vanderbilt University, USA). The primary outcome was COVID-19 in-hospital mortality within the 30 days following hospital admission. We also explored EVs quantification in The SAACOAG cohort. This is a prospective, single-center cohort including consecutive adult patients admitted for acute type A aortic dissection and undergoing urgent surgery, with systematic blood sampling performed at admission before surgery. The study was approved by the national ethics committee (Comité de Protection des Personnes CPP Sud-Est VI, Ref ID RCB 2019-A00645-52) and registered on ClinicalTrials.gov (NCT05149261).

### Platelet-poor plasma processing and handling for extracellular vesicles

Blood sampling was collected by venipuncture after having discard the first ml using 129 M trisodium citrate tubes as compatible with downstream analysis and was performed at hospital admission (within 48h following hospitalization) to be processed as quick as possible while taking care of avoiding excessive agitation and low temperatures of samples. Platelet-poor plasma (PPP) was obtained after a double centrifugation at room temperature at 2500xg for 15 min, in accordance with the recommendations of the GFHT and ISTH for hemostasis assays (33–36) and following MISEV2023 guidelines (22). Contamination/co-isolation of blood EVs by platelets, lipoproteins, hemolysis products, and a host of soluble/aggregated proteins including RNPs was not assessed but after visual inspection of plasma to exclude any hemolysis was systematically performed prior to samples storage of 500 microliters aliquots at –80 °C until analysis. Samples were submitted to one single freeze-thaw cycle prior being defrosted at 37 °C for 2 minutes on the day of analysis and centrifuged twice at 2000xg for 10 min. If necessary and based on counting, EVs concentration was adjusted for downstream analysis by dilution with 0,22 um filtered PBS to reach 100 ul of required solution and immediately analyzed.

### EV titration and measurement by Videodrop and nanoparticle-tracking analysis

Videodrop and Nanoparticle-Tracking Analysis (NTA) analyses were performed on a subset of 48 patients (27 critical, 21 non-critical). These analyses require fresh or rapidly thawed plasma samples, and due to technical constraints (instrument availability and sample instability). Videodrop (Myriade, France), a custom microscope that records in real time and uses the interference phenomenon to detect the light scattered by individual nanoparticules in solution. Using this interferometric signal, EVs are automatically detected and tracked to compute concentration and hydrodynamic parameter. Videodrop films in real time all particle between 80 nm and 1 µm in a drop and a concentration range at 10^8^ to 10^11^ particles per mL. When EVs concentration exceeded the detection limit, samples were diluted with filtered PBS (Gibco™, Fisher Scientific) 1/2, 1/4 or 1/10. The Videodrop protocol was fixed with the following parameter acquisition: number of frames 100, averaging frames 100, acquisition time 7.15 msec, exposure time 0.9 msec. NTA was performed using Zetaview (PMX120) (37), an interferometry technology device, as well as the Particle Matrix software, using the following parameters: laser wavelength 488 nm, pH 7.0, room temperature around 22 °C conductivity around 15000.00 µS/cm sensed, laser wavelength 488 nm and temperature room, pH 7.0. Isolated EVs were diluted in particle-free 0.22 µm filtered sterile PBS, held in a syringe of 1mL, and injected into the Nanosight tracking chamber. Capturing options were set to 60sec at a camera gain of 560 (LM10). For measurements with the device, samples diluted in 0.22 µm filtered sterile PBS were injected using a syringe pump and particles were tracked under constant flow. When the EV concentration exceeded the detection limit, samples were diluted with filtered PBS to 1/1000, 1/2000 or 1/5000. Each EV samples was measured for 60sec, three times. For background measurements, 0.22 µm filtered sterile PBS was injected into the chamber and measured using the same conditions. NTA detects all particle between 10 nm and 1000 nm in a concentration range of 10^6^ to 10^9^ particles per mL.

### MACSPlex analysis

MACSPlex EV surface marker analysis was performed on 80 samples (40 critical, 40 non-critical). The MACSPlex assay is designed to capture EVs with beads quoted with capture antibody as a first step, so that soluble fraction can bind to the capture antibody. However, the quantification in the second step uses tetraspanin staining so only EVs are quantified. MACSPlex analysis was performed using the MACSPlex Exosome Kit Immuno-Oncology, human (Miltenyi Biotec, Bergisch-Gladbach, Germany), which detects 37 exosomal surface epitopes plus two isotype controls. EV-containing samples were processed as follows: 120µL plasma containing EVs from each samples were diluted with 15µL MACSPlex EV Capture Beads. After overnight incubation at room temperature protected from light in an orbital shaker (450 rpm), 500 μL MACSPlex Buffer was added to each tube and centrifuged at room temperature at 3000g for 5 min. The supernatant was aspirated and 5 μL MACSPlex Exosome Detection Reagent for all 37 epitopes were added to each tube followed by incubation for 1 h at room temperature protected from light in an orbital shaker (450 rpm). The washing step was repeated and 500 μL MACSPlex Buffer was added to each tube followed by incubation for 15 min at room temperature protected from light in an orbital shaker (450 rpm). Samples were centrifuged and the supernatant aspirated, leaving about 150 μL in the tube. Following final bead incubation and washing, samples were transferred to flow cytometry tubes for acquisition without further filtration to avoid bead loss. Flow cytometric analysis was carried out on an Aurora Spectral Cytometer (Cytek ^®^ Biosciences, USA) and analysis was done with FlowJo software (v10.7.1, Treestar, USA). All markers were simultaneously analyzed. For the interpretation of results, we proceeded in several steps. First, the Mean Fluorescence Intensity (MFI) of the blank control was subtracted from the MFI of each epitope. Then, the result obtained was normalized with the MFI of the isotype *controls* (REA control isotype for recombinant human IgG and mIgG1 control isotype for mIgG). For this, a ratio MFI (%): MFI of EV-specific epitopes/MFI of corresponding control isotype was calculated. We did not use the commonly used strategy to analyze MACSPlex since to our opinion several technical and conceptual aspects can influence the analytical approach. Indeed, The MACSPlex bead-based assay captures EVs via epitope-specific antibodies, but quantification is performed through tetraspanin detection (CD9/CD63/CD81) in the detection step. Therefore, the signal obtained already reflects EV-bound tetraspanin-positive particles rather than soluble protein fractions. For this reason, we normalized all MFI values to their respective isotype controls (MFI ratio), which corrects for background and inter-sample variability. This specific MFI-ratio approach, based on normalization with the corresponding isotype controls, provides a very accurate representation of epitope-specific signals. By comparing each epitope directly to its isotype background, it enhances the ability to detect subtle variations in antibody binding. In contrast, normalizing to the mean MFI of tetraspanins can artificially smooth or mask differences, since the tetraspanin signal itself may vary across samples. Using the isotype-based ratio therefore offers a more sensitive and reliable way to distinguish true epitope-specific changes.

### Statistical analysis

Continuous data were expressed as medians (interquartile ranges [IQRs]) with categorical data as proportions. N For between-group comparison, we used the Mann-Whitney U test to determine differences in subset distribution of the two groups. To estimate the ability of biomarkers to predict in-hospital mortality, we used receiver operator characteristics (ROC) analysis. We estimated the area under the curve (AUC) and its 95% confidence interval (CI). The probability for survival was done by Kaplan-Meier method with log-rank tests (Mantel-Cox) univariate analyses. Furthermore, to assess the association between biomarkers levels and comorbidities and residual symptoms, we performed unadjusted logistic regression which was expressed as Odds ratio (OR) 95% confidence interval [CI]. In addition, we used the logistic regression model to investigate the relationships between the increase in the EV surface epitopes CD86 surface and mortality (both treated as categorical variable using the median as cut-off) adjusted for CRP levels. The comparison of the reference method (NTA) to a new method (Videodrop) used the Bland-Altman method. This method calculates the mean difference between two methods of measurement, and 95% limits of agreement as the mean difference. Bland and Altman advocated the use of a graphical method to plot the difference scores of two measurements against the mean for each subject. Bland-Altman is a represented as a plot of the mean difference (bias) with ±1.96 SD limits of agreement. We have applied a Bonferroni correction for multiple comparisons across the five primary MACSPlex-derived markers analyzed in the ROC and Kaplan-Meier survival curves. As such, all p-values have been adjusted to a significance threshold of p < 0.01 (i.e., 0.05/5) instead of 0.05. We have not applied this correction to the heatmap analysis, as it was designed as an exploratory and hypothesis-generating tool to screen broader epitope patterns. The statistical significance was accepted for *p* < 0.05. All statistical analyses were performed using the Stata software package (Stata, version. 12.0; StataCorp) and GraphPad Prism version 9.0.0 for Windows (GraphPad Software, USA).

## Results

### Population description

Briefly, the COVID-19 cohort consisted of 80 hospitalized patients (57 males and 23 females) with a median age of 64 years (age range: 35 to 98). Patients’ clinical characteristics are summarized in **Table 1**. Based on clinical severity at admission, 40 patients were classified as critical and were admitted in ICUs; 40 patients were classified as non-critical and were admitted in medical wards. As expected, critical patients had a higher body mass index (BMI median 29.1 kg/m² IQR [26.10-31.90], *p* = 0.004), while age and sex ratios did not significantly differ between groups (p=0.208. for age and p = 0.087 for sex ratio). Most patients of both groups had underlying co-morbidities of which the most frequent were hypertension (55%), hyperlipidemia (26.3%) and diabetes (27.5%). Regarding lab tests at admission, critical patients displayed significantly higher inflammatory and coagulation activation markers: CRP level (median 231.3 mg/L IQR [149.5-316.7]), fibrinogen (median 7.2 g/L IQR [6.2–8.5]), D-dimer (median 4137 µg/L IQR [2068–8263]), fibrin monomers (median 7.2 µg/mL IQR [6.2-8.5]) in comparison with non-critical patients (CRP: 84.4 mg/L [27.4–121.0], *p* < 0.001; fibrinogen 5.9 g/L [4.9–6.9], *p* < 0.001; D-dimer: 1435 µg/L [830–2463], *p* < 0.001; fibrin monomers: 5.9 µg/mL [4.9–6.9], *p* = 0.009**;****Table 1**). Furthermore, critical patients displayed significantly higher troponin, a cardiac marker (median 173.2 ng/L IQR [14.2–138.3]) in comparison with non-critical patients (38.9 ng/L [4.6–17.5], *p < 0.001*). Concerning blood cell count, hemoglobin level and lymphocyte count were significantly lower in critical patients (hemoglobin median 10.7 g/dL IQR [8.9 – 12.3]); lymphocytes median 0.8 G/L IQR [0.4 – 1.1]) compared to non-critical patients (hemoglobin median 12.6 g/dL IQR [11.5 – 14], p<0.001; lymphocytes median 1.2 IQR [0.7-1.3], p = 0.01). A significant difference was also found regarding neutrophil count, with neutrophilia in critical patients (neutrophils median 12.4 G/L IQR [8.8 – 17.5]), not found in non-critical patients (neutrophils median 4.9 IQR [3.7 – 6.1], p <0.001). As expected, a higher mortality level and a more widely used intubation were reported in critical patients compared to non-critical patients (**Table 1**).

**Table 1:** Demographic, clinical and biological characteristics of COVID-19 patients at admission.

Label of the data	Non critical COVID-19	Critical COVID-19	P-value
Demographic data			
Age, years - median [IQR]	65.9 [55 – 73.8]	60.4 [52 – 71.8]	0.208
BMI, kg/m² [IQR]	26.1[23.2 – 29.0]	29.1 [26.1 – 31.9]	**0.004**
Male sex, n(%)	25 (62.5)	32 (80)	0.087
Female sex, n (%)	15 (37.5)	8 (20)	
Comorbidities			
Diabetes, n (%)	9 (22.5)	13 (32.5)	0.323
Hypertension, n (%)	22 (55)	22 (55)	0.996
Dyslipidemia, n (%)	8 (20)	13 (32.5)	0.209
History or active malignancy, n (%)	7 (17.5)	2 (5)	0.080
Biological results			
Hemoglobin, g/dL – median [IQR]	12.6 [11.5–14]	10.7[8.9–12.3]	**<0.001**
Platelets, G/L – median [IQR]	261[184–312]	278 [182–356]	0.477
Neutrophils, G/L – median [IQR]	4.9[3.7–6.1]	12.4[8.8–17.5]	**<0.001**
Lymphocytes, G/L – median [IQR]	1.2[0.7–1.3]	0.8[0.4–1.1]	0.011
Monocytes, G/L – median [IQR]	0.6[0.4–0.7]	1.5[0.3–0.9]	0.571
CRP, mg/L – median [IQR]	84.4[27.4–121.0]	231.3[149.5–316.7]	**<0.001**
Fibrinogen, g/L – median [IQR]	5.9[4.9–6.9]	7.2[6.2–8.5]	**<0.001**
D-dimer, µg/L – median [IQR]	1435[830–2463]	4137[2068–8263]	**<0.001**
Fibrin Monomer, µg/mL – median [IQR]	5.9[4.9–6.9]	7.2[6.2–8.5]	**0.009**
Troponin, ng/L – median [IQR]	38.9[4.6–17.5]	173.2[14.2–138.2]	**<0.001**
Outcomes (%)			
Intubation, n (%)	1 (2.5)	40 (100)	**<0.001**
Mortality, n (%)	1 (2.5)	19 (47.5)	**<0.001**

IQR, interquartile range; BMI, body mass index; CRP, C-reactive protein.

Statistically significant p-values are shown in bold.

### Circulating large-EVs are increased in critical COVID-19 by Videodrop interferometry method

**Figure 1:**
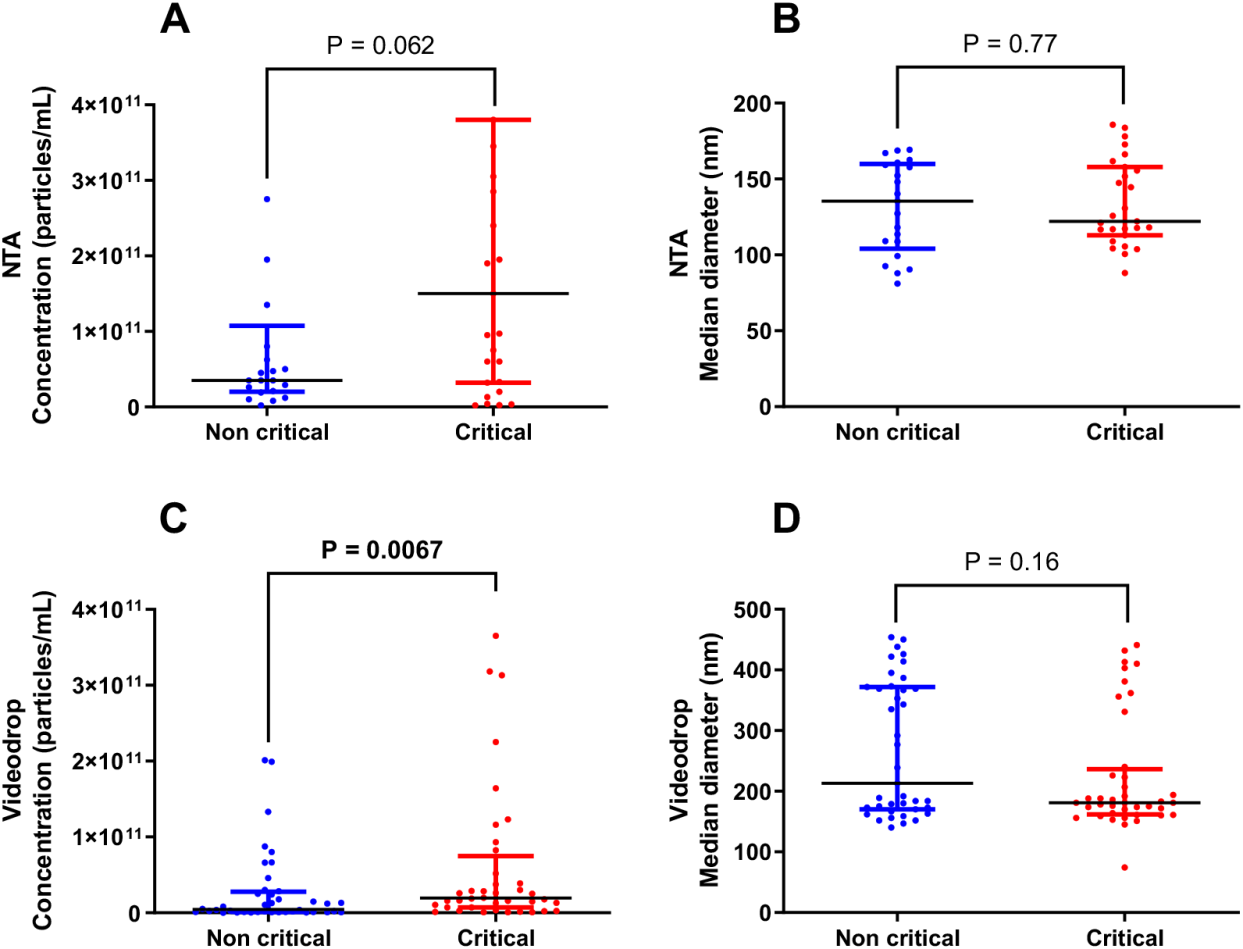
Characterization of concentration and size of EVs by two different interferometry methods. EVs, extracellular vesicles; NTA, Nanoparticle Tracking Analysis. **:p<0.01 **(A)** EV concentration (particles/mL) in critical and non-critical COVID-19 patients with NTA technology (Malvern Panalytical). **(B)** EV size (median diameter, nm) in critical and non-critical COVID-19 patients with NTA technology. **(C)** EV concentration (particles/mL) in critical and non-critical COVID-19 patients with Videodrop technology (Myriade, France). **(D)** EV size (median diameter, nm) in critical and non-critical COVID-19 patients with Videodrop technology.

**Figure 2:**
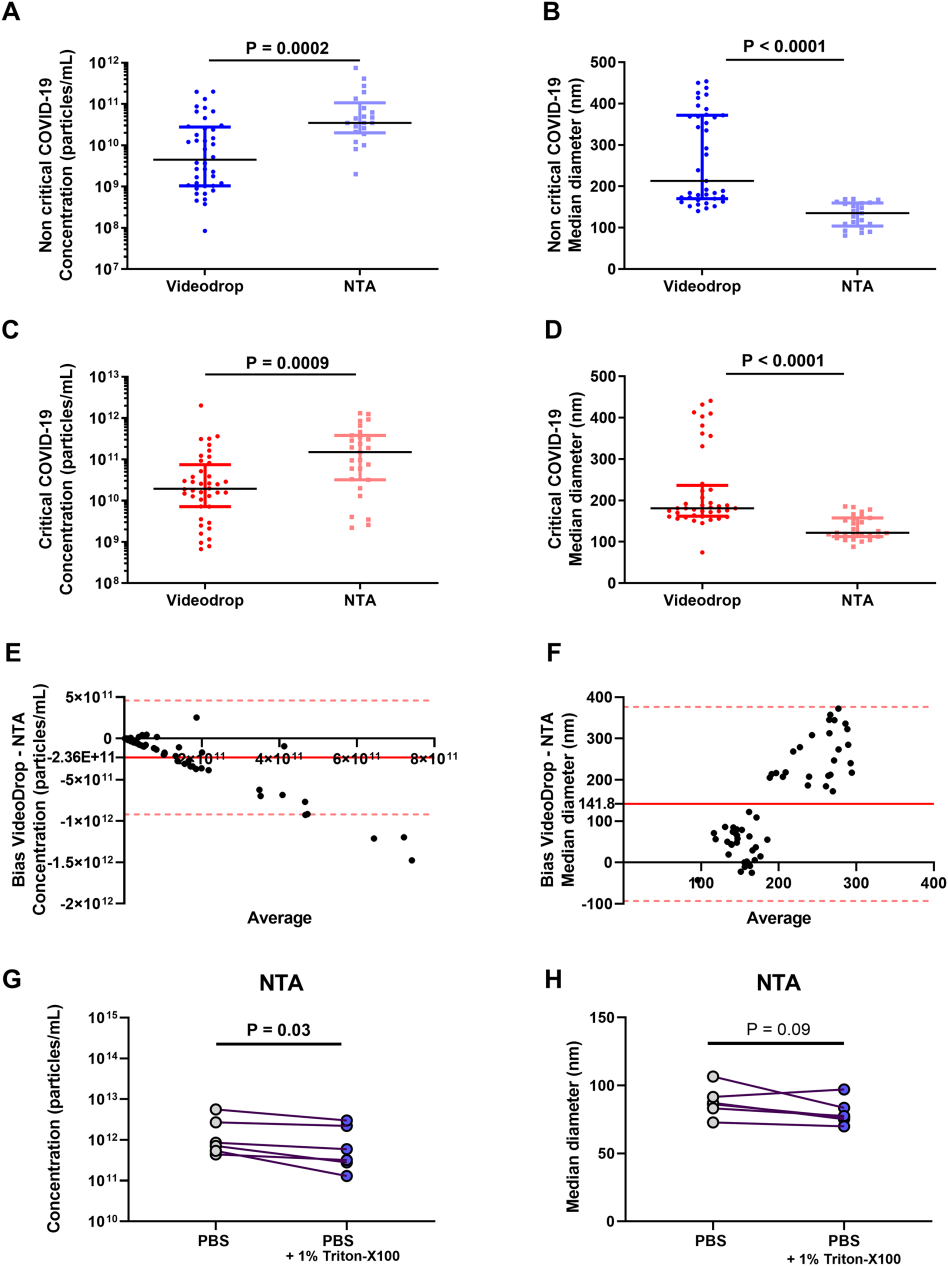
Comparison of two different interferometry methods: NTA (Malvern Panalytical) and Videodrop (Myriade, France) in COVID-19. EVs, extracellular vesicles; NTA, Nanoparticle Tracking Analysis. **p < 0.01, ***p<0.001 **(A)** EV concentration (particles/mL) in non-critical COVID-19 patients with Videodrop and NTA technologies. **(B)** EV (median diameter, nm) in non-critical COVID-19 patients with Videodrop and NTA technologies. **(C)** EV concentration (particles/mL) in critical COVID-19 patients with Videodrop and NTA technologies. **(D)** EV (median diameter, nm) in non-critical COVID-19 patients with Videodrop and NTA technologies. **(E)** Difference diagram Bland-Altman of EV concentration (particles/mL) of all COVID-19 patients’ cohort. **(F)** Difference diagram Bland-Altman of EV size (median diameter, nm) of all COVID-19 patients’ cohort. **(G)** Effect of Triton X-100 detergent treatment on overall particle concentration. **(H)** Effect of Triton X-100 detergent treatment on the size distribution.

**Figure 3:**
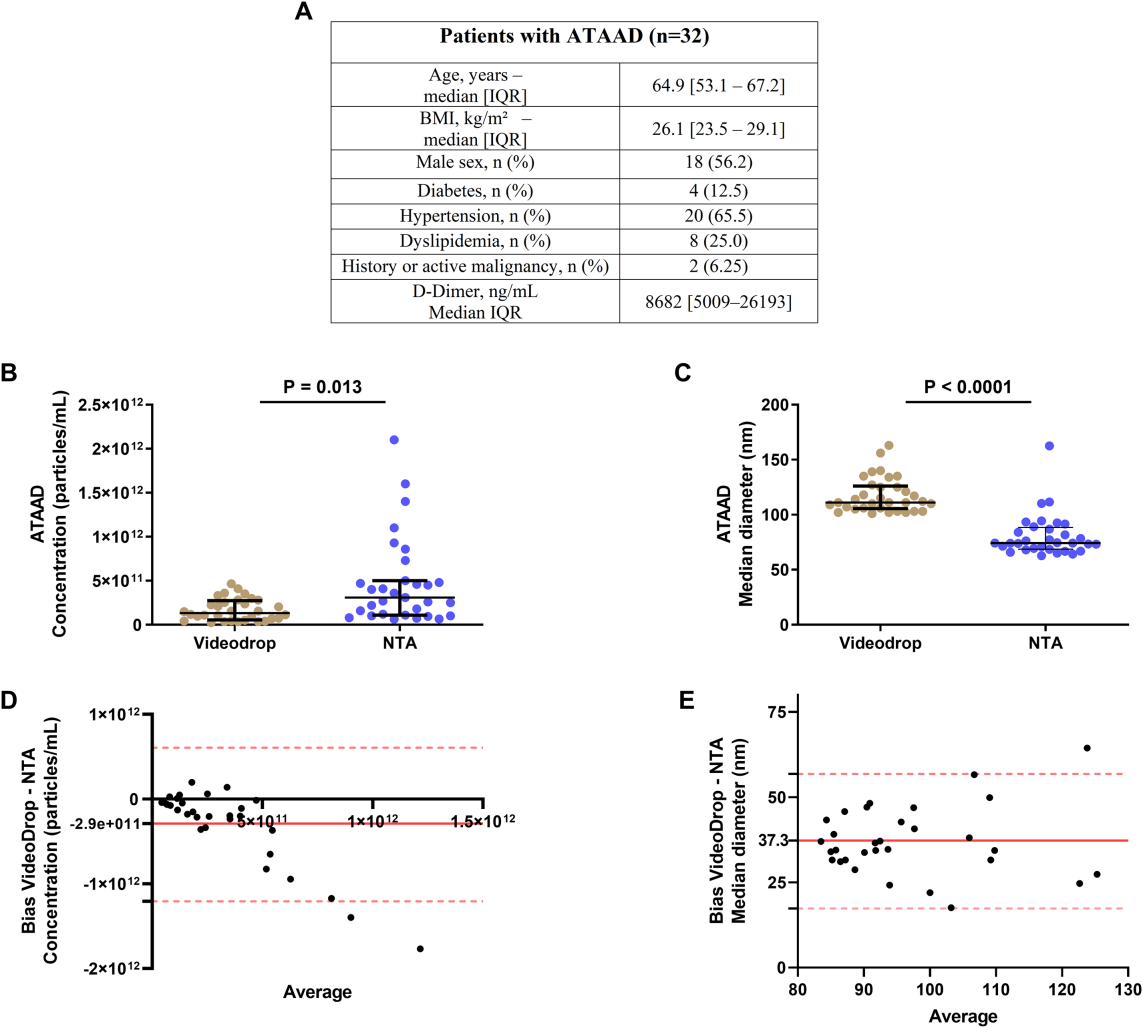
Comparison of two different interferometry methods: NTA (Malvern Panalytical) and Videodrop (Myriade, France) in acute type A aortic dissection. EVs, extracellular vesicles; NTA, Nanoparticle Tracking Analysis. **p < 0.01, ***p<0.001 **(A)** Acute type A aortic dissection patients’ description. **(B)** EVs concentration (particles/mL) between Videodrop and NTA technologies. **(C)** EVs Size distribution between Videodrop and NTA technologies. **(D)** Difference diagram Bland-Altman of EV concentration (particles/mL) of Acute type A aortic dissection patients’ cohort. **(E)** Difference diagram Bland-Altman of EV size (median diameter, nm) of Acute type A aortic dissection patients’ cohort.

EV size and concentration data were available for 48 out of the 80 patients (27 in the critical group and 21 in the non-critical group) due to the slow measurement process and the time required, which affected sample stability. Using NTA technology, median EV concentrations were similar between critical patients (1500x10^8^ particles/mL, IQR 320x10^8^ – 3800x10^8^) and non-critical patients (350x10^8^ particles/mL, IQR 200x10^8^– 1075x10^8^, p=0.06, **Figure 1A**). The median EV size also showed no significant difference between critical (122 nm, IQR 113-158) and non-critical patients (135 nm, IQR 104-160, p=0.78, **Figure 1B**). In contrast, Videodrop analysis revealed a significantly higher circulating EV concentration in critical patients (median 194x10^8^ EVs/mL, IQR 71.9x10^8^ – 748x1^8^) compared to non-critical patients (median 44.8x10^8^ EVs/mL, IQR 10.5x10^8^ – 277.5x10^8^, p=0.007, **Figure 1C**). However, the median EV size diameter did not differ significantly between critical (181 nm, IQR 162-237) and non-critical patients (213 nm, IQR 170-372, p=0.16, **Figure 1D**). Subsequently, EV size and concentration were compared between the two interferometry methods. In non-critical patients, NTA showed a higher EV concentration but a lower median diameter compared to Videodrop (p<0.001, **Figures 2A, B**). Similar findings were observed in critical patients (p=0.001, **Figures 2C, D**). Lastly, a Bland-Altman plot comparison of the two methods across the entire cohort indicated a measurement discrepancy between them, suggesting a potential bias in analysis. In order to better discriminate EVs from potential non-vesicular contaminants such as lipoproteins or protein aggregates, we performed additional control experiments using Triton X-100 detergent treatment, as shown in **Figures 2G, H**. In these experiments, EV-enriched plasma samples from COVID-19 patients were first quantified by NTA and then treated with 1% Triton X-100 (by diluting samples 9:1 with 10% Triton-X100), incubated for 30 minutes at room temperature, and then reanalyzed using NTA. Triton X-100 is known to disrupt lipid bilayer membranes while leaving detergent-resistant particles such as protein aggregates and lipoproteins largely intact. As expected, we observed a significant reduction in overall particle concentration following detergent treatment (**Figure 2G**), while the size distribution of the detected particles remained unchanged (**Figure 2H**). This is consistent with the fact that our measurement threshold excludes particles smaller than 40 nm, and thus likely does not capture disrupted vesicles. The observed decrease in particle count supports the interpretation that the majority of particles present before treatment were membrane-bound EVs, rather than detergent-insensitive entities such as lipoproteins or protein aggregates. We finally performed a complementary experiment in a new cohort of 30 patients with acute type A aortic dissection (ATAAD), a condition also characterized by systemic inflammation and coagulopathy. These patients are non-infectious conditions however they have a coagulopathy (38) as previously described in covid19 (39). In these samples, we performed both Videodrop and NTA analysis. As shown in **Figure 3**, we found the same discrepancies between size and concentration that the one observed in COVID-19.

### EV surface epitope expression is associated with clinical admission severity and mortality of patients with COVID-19

**Figure 4:**
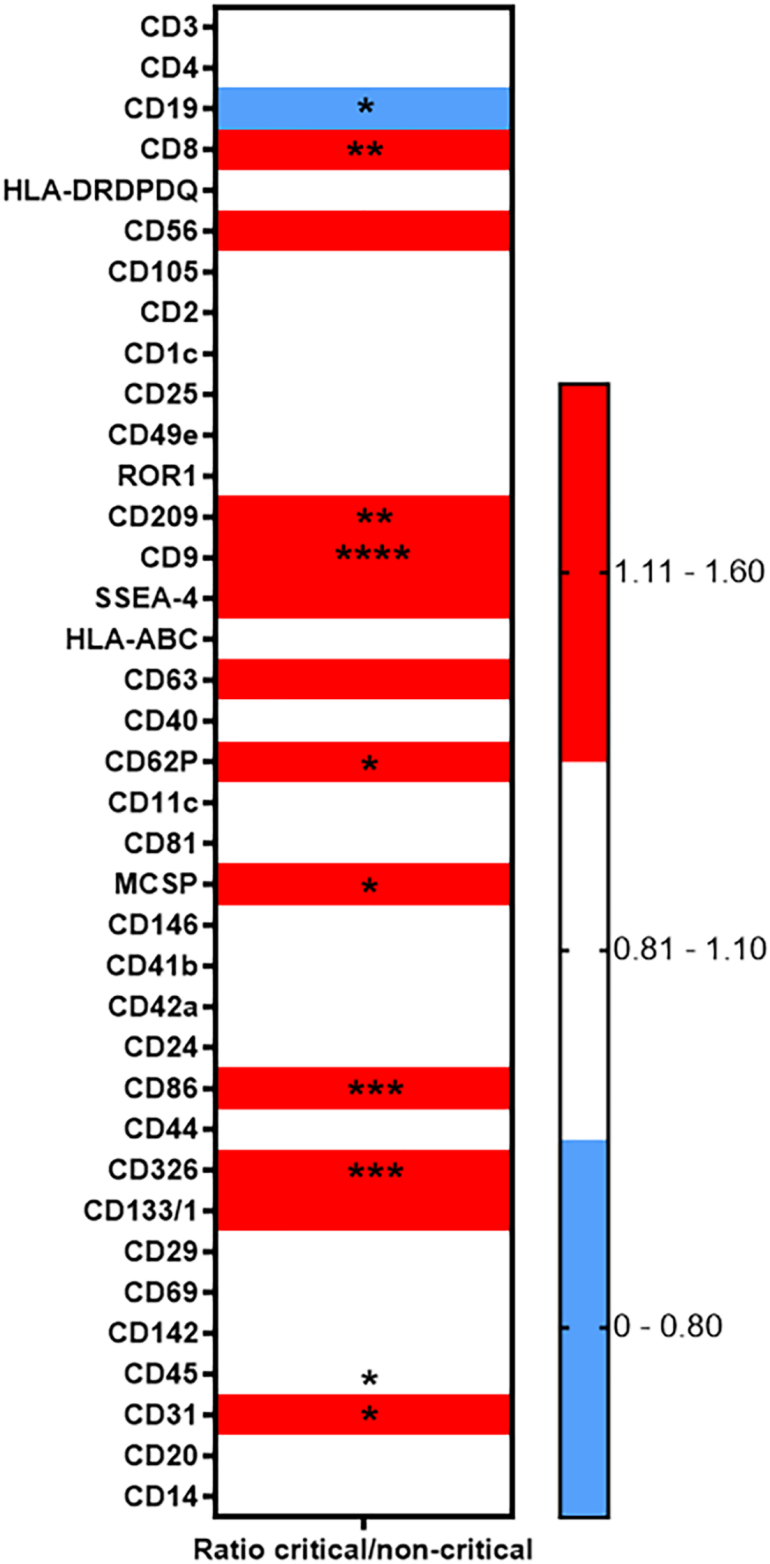
MACSPlex exosomes heatmap - EV surface epitope expression. Analysis of 37 surface epitope expression and two surface control isotypes by bead-based multiplex flow cytometry (MACSPlex) method. Heatmap represents the ratio obtained in Critical versus Non-Critical groups for each epitope. The color code is as follows: blue for decreased expression, white for same expression and red for increased expression. Some EV surface markers were correlated with COVID criticality, as indicated by *p< 0.05, **p<0.01, ***p<0.001 and ****p<0.0001, (Mann-Whitney test). EVs, extracellular vesicles; ELISA, enzyme-linked immunosorbent assay.

**Figure 5:**
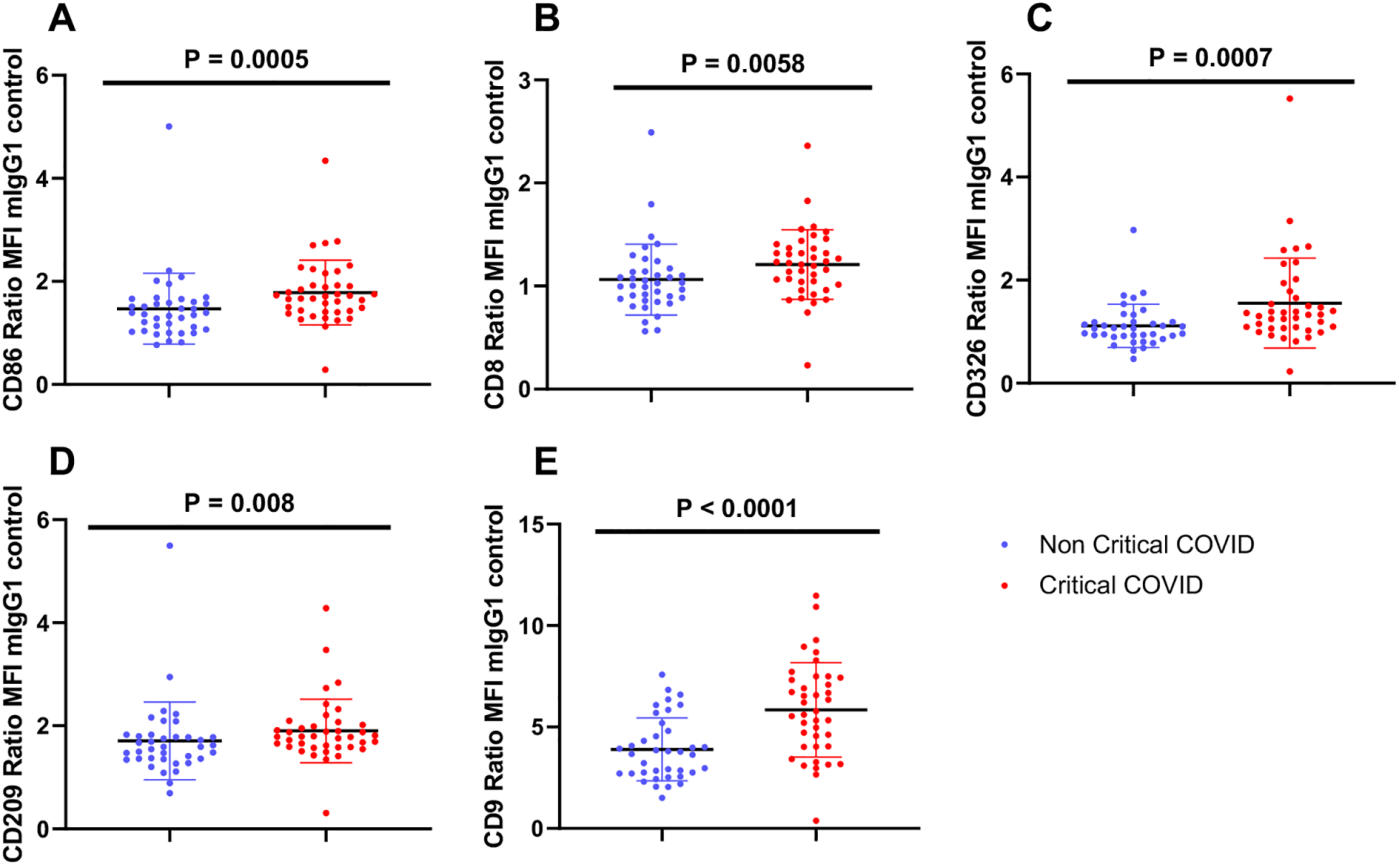
Representation of MACSPlex analysis. Results for each epitope are reported as ratio MFI. Surface epitope levels were referenced to EV-specific epitopes by subtracting the respective fluorescence values of blank control from MFI values for individual epitopes and normalizing them for isotype MFI value controls. EV: extracellular vesicles; MFI: mean fluorescence intensity. **(A)** Ratio MFI of CD86-EV in non-critical and critical COVID-19 patients, p<0.001. **(B)** Ratio MFI of CD8-EV in non-critical and critical COVID-19 patients, p<0.001. **(C)** Ratio MFI of CD326-EV in non-critical and critical COVID-19 patients, p<0.001. **(D)** Ratio MFI of CD209-EV in non-critical and critical COVID-19 patients, p = 0.004. **(E)** Ratio MFI of CD9-EV in non-critical and critical COVID-19 patients, p<0.001.

**Figure 6:**
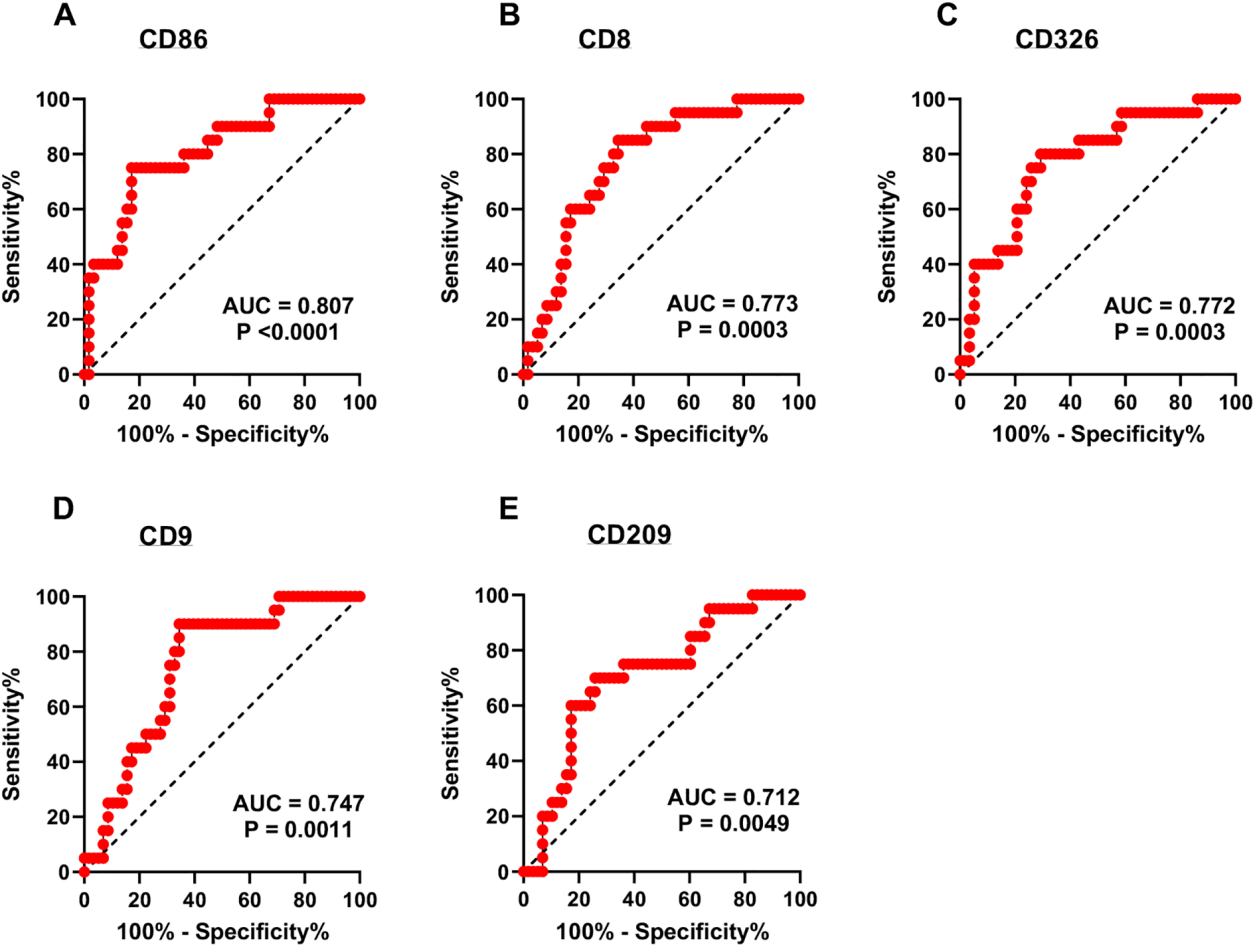
ROC curves to evaluate biomarkers of interest for in-hospital mortality. **(A)** ROC curves of all patients (critical and non-critical COVID-19) for EVs CD86+, AUC = 0,807. **(B)** ROC curves of all patients (critical and non-critical COVID-19) for EVs CD8+, AUC = 0,773. **(C)** ROC curves of all patients (critical and non-critical COVID-19) for EVs CD326+, AUC = 0,772. **(D)** ROC curves of all patients (critical and non-critical COVID-19) for EVs CD9+, AUC = 0,747. **(E)** ROC curves of all patients (critical and non-critical COVID-19) for EVs CD209+, AUC = 0,712. ROC, receiver operating characteristic; EVs, extracellular vesicles; AUC, area under the curve. We have applied a Bonferroni correction for multiple comparisons across the five primary MACSPlex-derived markers analyzed in the ROC curves. As such, all p-values have been adjusted to a significance threshold of p < 0.01 (i.e., 0.05/5) instead of 0.05.

**Figure 7:**
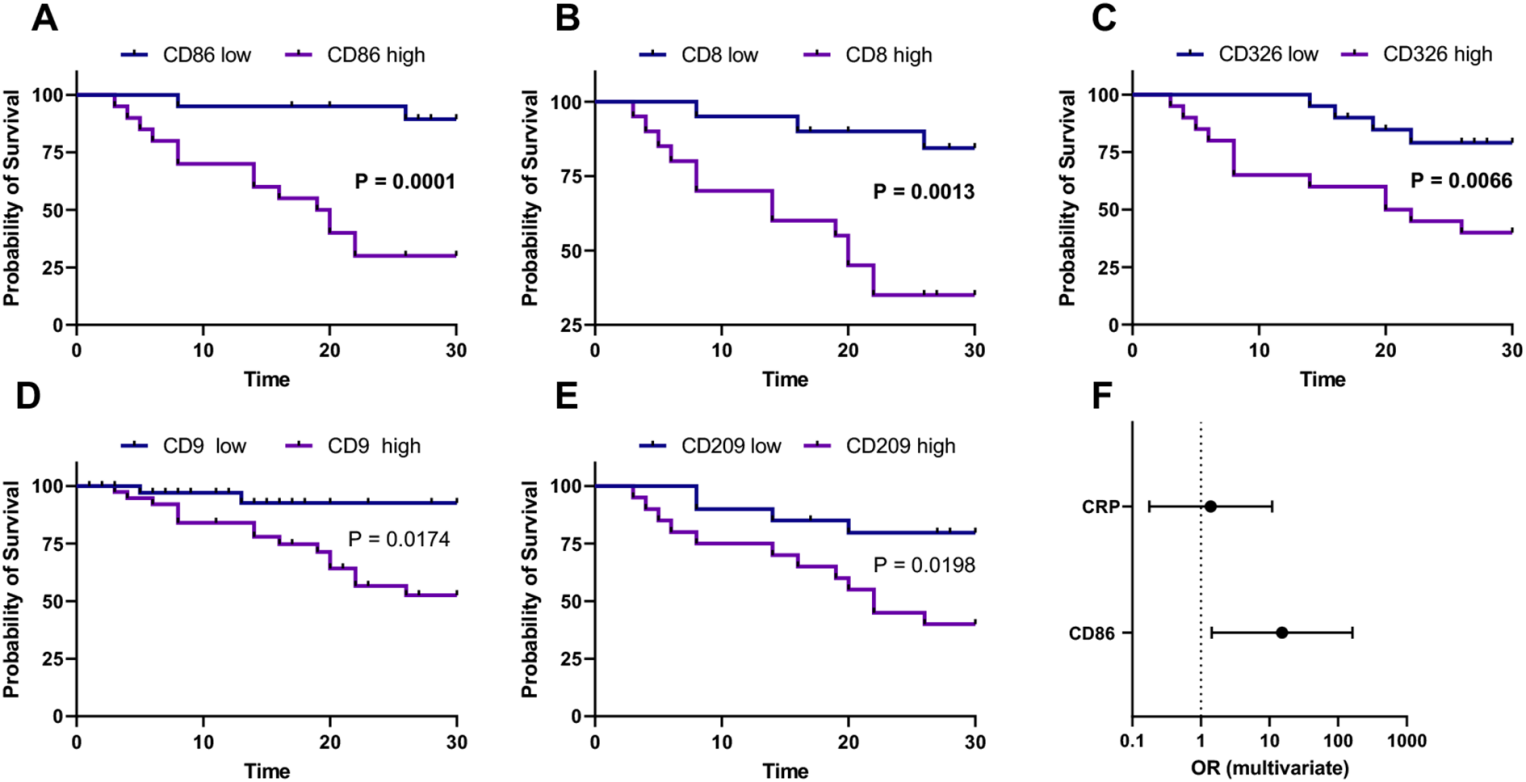
Kaplan-Meier survival analysis for critical and non-critical COVID-19 patients with, stratified according EV surface expression intensity of CD86, CD8, CD329, CD9 and CD209. Using whole cohort median value for each epitope of interest, the two subgroups showed a significant difference in survival rate following stratification according to median epitopes expression. EVs, extracellular vesicles. We have applied a Bonferroni correction for multiple comparisons across the five primary MACSPlex-derived markers analyzed in the Kaplan-Meier survival curves. As such, all p-values have been adjusted to a significance threshold of p < 0.01 (i.e., 0.05/5) instead of 0.05. **(A)** Kaplan-Meier for EVs surface expression intensity of CD86, p<0,001. **(B)** Kaplan-Meier for EVs surface expression intensity of CD8, p=0,0013. **(C)** Kaplan-Meier for EVs surface expression intensity of CD326, p 0,0066. **(D)** Kaplan-Meier for EVs surface expression intensity of CD9, p 0, 0174. **(E)** Kaplan-Meier for EVs surface expression intensity of CD209, p 0,0198. **(F)** Cox proportional hazard analysis of CD86 median for in-hospital mortality adjusted for CRP levels.

The heatmap diagram in **Figure 4** illustrates the global analysis by showing the ratio of critical to non-critical cases, calculated by dividing the MFI of the critical group by the MFI of the non-critical group for each epitope. Compared to non-critical patients, the EV surface epitopes CD86 (p < 0.001, **Figure 5A**), CD8 (p < 0.001, **Figure 5B**), CD326 (p < 0.001, **Figure 5C**), CD209 (p = 0.009, **Figure 5D**), and CD9 (p < 0.001, **Figure 5E**) were significantly elevated in critical patients. In contrast, CD19 levels were significantly lower in critical patients (p = 0.017). Regarding in-hospital mortality outcomes, EV surface epitopes CD8, CD86, CD9, CD326, CD209, CD133, CD29, CD63, and CD24 were significantly higher in survivors compared to non-survivors (p < 0.001, p < 0.001, p < 0.001, p < 0.001, p = 0.004, p = 0.013, p = 0.013, p = 0.011, p = 0.048, respectively). Other assessed EV epitopes did not show significant differences between non-survivors and survivors. To evaluate the discriminatory ability of these biomarkers for predicting in-hospital mortality, ROC curves were generated. The four surface epitopes with the highest predictive ability for mortality were CD86 (AUC 0.807, 95% CI 0.70 - 0.92, p < 0.001, **Figure 6A**), CD8 (AUC 0.773, 95% CI 0.66 – 0.88, p < 0.001, **Figure 6B**), CD326 (AUC 0.772, 95% CI 0.66 – 0.89, p < 0.001, **Figure 6C**), CD9 (AUC 0.747, 95% CI 0.63 – 0.86, p = 0.001, **Figure 6D**), and CD209 (AUC 0.712, 95% CI 0.58 – 0.84, p = 0.005, **Figure 6E**). **Figure 7** presents the Kaplan-Meier survival analysis for critical and non-critical COVID-19 patients, stratified according to their EV surface expression levels of CD8, CD86, CD326, and CD209. When stratifying by the whole cohort median value for each epitope, significant differences in survival rates were observed. Specifically, survival was significantly different based on median CD86 expression (p < 0.001, hazard ratio: 0.097, 95% CI 0.036-0.264, **Figure 7A**), median CD8 expression (p = 0.001, hazard ratio: 0.167, 95% CI 0.062-0.450, **Figure 7B**), CD326 expression (p = 0.0066, hazard ratio: 0.252, 95% CI 0.094-0.678, **Figure 7C**), CD9 expression (p = 0.0174, hazard ratio: 0.175, 95% CI 0.067-0.457, **Figure 7D**), and CD209 expression (p = 0.0198, hazard ratio: 0.272, 95% CI 0.102-0.729, **Figure 7E**). Thus, CD86 emerged as the most critical marker for assessing the severity and mortality of COVID-19 infection, prompting a multivariate analysis. The predictive power of the CD86 median for in-hospital mortality was validated using a Cox proportional hazard analysis, adjusted for CRP levels. This analysis, which dichotomized the CD86 MFI ratio based on the median, highlighted the significance of CD86 as an independent predictor, regardless of the level of inflammation (Odds ratio: 15.20, 95% CI 1.43-160.9, p = 0.03, **Figure 7F**).

## Discussion

Our study shows that EVs are associated with disease severity and in-hospital mortality in COVID-19 and may serve as potential biomarkers, although causality remains to be established. By utilizing two different interferometry methods, NTA and Videodrop, we were able to investigate the concentration and size of EVs in critical and non-critical COVID-19 patients, revealing important differences in their profiles. The results indicated that while NTA did not show a significant difference in EV concentration between critical and non-critical patients, Videodrop analysis revealed a notably higher concentration of circulating EVs in critical patients. This discrepancy highlights the sensitivity of different measurement techniques and suggests that Videodrop may be better suited for detecting subtle variations in EV concentrations in severe COVID-19 cases.

Circulating EVs have been recently reported in COVID-19 infection as a potential biomarker of this infection (5–15). Converging evidence designate EVs as crucial mediators of intercellular communication, stress signal transduction, and inflammatory responses (40), for example, during sepsis (41). While some studies showed that EV levels did not differ between patients with severe and non-severe COVID-19 (42), we report several new findings, evidencing subpopulations of EVs showing an immuno-inflammatory epitope signature upregulated in critical patients. These EVs were associated with in-hospital mortality, suggesting that they might play a key role in the progression of disease. The study of the concentration of EVs and their size is of interest in numerous studies. Some preclinical studies demonstrated a role of large EVs as functional inflammatory mediators of sepsis. Monnamonn et al. revealed a higher EV concentration in septic shock patients compared to infected patients without septic shock. In addition, in septic shock patients, EV concentration decreased over time as opposed to infected patients (43). About EV size, we showed an increase of large EVs in the most severe patients, at least with one of the two methods used. Furthermore, there was an increase in EV count in these critical patients. Concerning the discrepancy between the two methods used, Videodrop is based on interferometric light microscopy while NTA shows Brownian motion (44). Algorithms used to analyze data are different. NTA uses particle-tracking algorithms to calculate the movement of vesicles and infer their size. Videodrop uses light interference measurements that can be influenced by variations in refractive index and particle shape. The difference in extracellular vesicle size measurement between Videodrop and NTA therefore results from inherent differences in the measurement principles, experimental conditions, analysis algorithms, and technical aspects of each method. To achieve consistent and comparable results, it is important to understand and control these variables as much as possible. A previous study has recently highlighted the differences between Videodrop and NTA (45), with largest EVs only detected by Videodrop, indicating a lower sensitivity threshold compared to NTA. However, the performances of Videodrop were better than NTA for the determination of the EV concentration. Authors concluded that size measurements provided by NTA and Videodrop should be considered with caution, as they do not reflect the true geometrical size distribution. Based on these findings, NTA and Videodrop appear to be complementary for EV characterization. These finding might explain the discrepancy we describe regarding EV concentration according to severity in our cohort. Indeed, the critical patients showed a greater number of EVs compared to non-critical patients only using Videodrop but not NTA, that is in favor of an excess in large but not in small EVs. Therefore, the study of surface marker expression was performed in both small and large EVs. Several studies have demonstrated the utility of NTA in quantifying extracellular vesicle (EV) concentration and size in COVID-19, providing insights into disease severity. For example, Zipperle et al. reported a significant increase in particle concentration in ICU patients compared to healthy controls and non-ICU COVID-19 patients using NTA, although they did not observe time-dependent differences in particle size across patient groups (46). Their findings align with our own, where elevated EV concentrations correlated with severity, reinforcing the role of NTA as a valuable tool in characterizing the pro-inflammatory EV landscape in severe COVID-19. Similarly, Aharon et al. applied NTA in their analysis of EVs across mild, moderate, and severe COVID-19 cases, noting a shift toward smaller particles in severe patients, alongside increased expression of markers such as CD63 and ACE2 in EVs (47). These findings suggest dynamic remodeling of the EV population in response to disease progression, in agreement with the altered EV profiles we observed. Further supporting our data, Paes Leme et al. conducted an exploratory proteomic analysis of serum-derived EVs from COVID-19 patients, reporting no significant difference in EV concentration or size between mild and moderate/severe cases as measured by NTA, despite clear proteomic divergence (9). This highlights a potential limitation of NTA in capturing subtle functional shifts in EV populations, reinforcing the importance of complementary methods such as proteomics or flow cytometry. Finally, Dobra et al. demonstrated the predictive value of EV proteomic signatures in identifying patients at risk for post-COVID syndrome. Although their study did not identify significant concentration or size differences by NTA, it underscored how EV cargo – particularly complement-related proteins – may evolve independently of physical EV parameters (48). This is consistent with our observation that NTA provides essential but not exclusive insight into EV-mediated pathophysiology. Taken together, our results are consistent with and build upon the growing body of literature employing NTA to characterize EVs in COVID-19. While NTA offers reliable quantitative data on EV concentration and size, our findings and those of others support the conclusion that integrating NTA with phenotypic and functional analyses enhances its diagnostic and prognostic utility.

The MACSPlex has several advantages such as the simultaneous measurement of multiple analytes or even cytometry analysis, but it also had analytical limitations that may explain the differences in results compared to ELISA analyses used in other studies. MACSplex has a lower sensitivity than the ELISA technique and shows limitations as a single EV should express several markers included in the kit which may cause interference between analytes. Moreover, variations in EV concentration between samples, as well as background noise, could influence the results. For this reason, we opted to normalize our analysis by interpreting the ratio of MFI. One discrepancy exist in our results in contrast to findings of Burrello et al. (49). Their study identified several differentially expressed surface markers in patients with varying prognoses (CD49e, CD69, CD142, and CD45), with CD142-EV showing the strongest prognostic association, reporting a hazard ratio (HR) of 1.074 (95% CI: 1.032–1.119) in regression models. In contrast, our research highlights CD86, CD8, CD326, CD209, and CD9 as key predictors of disease severity and mortality, utilizing what appears to be the same MACSPlex Human Exosome Kit (Miltenyi) for analysis. However, a fundamental distinction between the two studies lies in the choice of biological material as the EV source: we adhered to the international recommendations by using double-centrifuged citrated plasma, whereas Burrello et al. based their analysis on serum (49), known to favor the generation of microvesicles from circulating blood cells. This methodological difference could significantly impact results and may present a critical limitation in their study.

Furthermore, our analysis of EV surface epitope expression identified several key markers associated with clinical severity and mortality. In particular, CD86, CD8, CD326, CD209, and CD9 were significantly elevated in critical patients, while CD19 was reduced. These findings suggest that specific EV subpopulations could be linked to the immune response and disease progression in COVID-19. During their biogenesis, EVs may selectively capture cell-specific proteins, lipids, RNAs, or even DNA, which may become a part of the EV membrane’s or cargo’s “molecular signature” (50, 51). Therefore, EVs represent a powerful tool to assess cellular proliferation and activation of cell population with a high degree of specificity. All the significantly modified surface markers are related to immuno-inflammation. From a mechanistic point of view, surface overexpression of EVs CD86, CD8, CD209 and CD9 in critical and non-survivor patients with COVID-19 is probably related to innate and adaptive immune system dysregulation that has been extensively described in COVID-19 (52). CD8 is mainly expressed on CD8+ T-cells, subpopulation of adaptive lymphocytes which play an important role in immunity to intracellular pathogens and tumors (53–55). Both CD86 (also known as B7-2) and CD209 are expressed by antigen-presenting cells, mainly dendritic cells and monocytes/macrophages, and bind as a ligand to costimulatory of all T cells during innate immune response (56, 57). Wang et al. showed that exhausted T cells released exosomes capable of altering proliferation and cytotoxic performance from healthy T cells (58). As expected in our cohort, we showed an increase of EVs with immuno-inflammatory markers in critical *vs* non-survivor patients (58). Moreover, it is known that during sepsis, lymphocytes are depleted by apoptosis, causing an immunosuppression (59). Thus, EVs bearing high levels of immune-inflammatory markers in critical and deceased patients could be the stigma of the dysregulated and impaired T-cell intense activation and subsequent lymphopenia causing collateral damage to the host tissue (60, 61). We also showed a surface overexpression of EVs CD326 in critical and non-survivor patients with COVID-19. CD326 or EpCAM is an epithelial cell adhesion molecule (62). In sepsis, Appiah et al. demonstrated that intestinal epithelial cells released CD326 (EpCAM+) EVs during sepsis (63). For COVID-19 infection, high levels of proinflammatory cytokines are associated with pulmonary inflammation and lung fibrosis (64, 65). In our study, the increase of CD326 in critical patients highlights this lung damage more important than in non-critical patients, as well as in patients with higher mortality.

The predictive value of CD86 emerged as especially noteworthy. Our multivariate analysis, adjusted for CRP levels, confirmed that CD86 is a robust independent predictor of in-hospital mortality, regardless of the inflammation level. This highlights the potential of CD86 as a critical biomarker for assessing the risk and guiding treatment strategies in COVID-19 patients. CD86, also known as B7-2, is a powerful T-cell co-stimulatory molecule expressed on the surface of antigen-presenting cells (23). This marker has already been studied during viral infection, particularly during MERS-COV-infection. Chu et al. showed higher surface expression of CD86 in MERS-COV-infection than in SARS-CoV-infection, by cytometry on Mo-DCs cells (66). They hypothesized a robust activation of T cells, which may lead to more destruction of infected pulmonary tissues and produce high levels of inflammatory cytokines. In line, Bobrie et al. showed that vesicles from dendritic cells carrying CD86 molecules can induce T cell activation (67). Therefore, the higher expression of CD86+ vesicles we describe could be a reflection of major T cell activation, which would lead to a cytokine cascade. Additionally, CD86+ EV expression was found to be most significantly relevant to criticality and mortality during COVID-19 infection in our cohort. This makes it a marker of interest for initial treatment of COVID-19 infection. Further mechanistic studies using EV uptake assays, co-culture with immune cells, and cytokine profiling will be essential to explore whether CD86^+^ EVs directly modulate immune responses.

This study has several limitations that should be acknowledged. First, while we used the MACSPlex Human Exosome Kit (Miltenyi) as a well-established method for EVs surface marker profiling, our findings would benefit from validation using alternative quantification techniques. Methods such as Western blot, ELISA, or nanoparticle flow cytometry could provide complementary evidence and further strengthen the reliability of our results. Due to resource constraints, such validation was not feasible within the scope of this study, but we recognize its importance and plan to incorporate additional methods in future research. Second, our analysis was conducted on a single-center cohort, which ensures consistency in patient management and sample processing but limits the generalizability of our findings. While expanding to a multicenter study would enhance the robustness and external validity of our results, logistical constraints prevented us from including data from multiple institutions. Future studies incorporating a broader, multicenter dataset will be crucial to confirm and expand upon our findings. Third, our study primarily focused on the diagnostic and prognostic significance of EV-associated surface markers, but their functional role remains unclear. For example, whether CD86+ EVs and other identified markers contribute to disease progression or immune modulation requires further investigation. Functional assays, such as *in vitro* experiments assessing the immunomodulatory properties of these EVs, would provide valuable mechanistic insights. While such experiments were beyond the scope of this study, they represent an important avenue for future research to better understand the biological role of EVs in COVID-19 pathophysiology. Moreover, epitope expression does not confirm cell of origin. CD86 and CD209 are expressed on both dendritic and monocyte-derived vesicles, which cannot be distinguished without additional co-detection (e.g., dual-labeled imaging flow cytometry), especially during inflammation.

All in all, our findings suggest that EVs, particularly those expressing CD86, could serve as critical biomarkers for both disease severity and mortality in COVID-19. These insights open new avenues for research into EV-targeted therapies and personalized treatment approaches, aiming to improve outcomes for patients with severe COVID-19. Further studies are needed to validate these findings in larger cohorts and to explore the underlying mechanisms linking EVs to COVID-19 pathogenesis.

## Data Availability

The raw data supporting the conclusions of this article will be made available by the authors, without undue reservation.
